# Adipose tissue dysfunction, inflammation, and insulin resistance: alternative pathways to cardiac remodelling in schizophrenia. A multimodal, case–control study

**DOI:** 10.1038/s41398-021-01741-9

**Published:** 2021-12-06

**Authors:** Emanuele F. Osimo, Mark Sweeney, Antonio de Marvao, Alaine Berry, Ben Statton, Benjamin I. Perry, Toby Pillinger, Thomas Whitehurst, Stuart A. Cook, Declan P. O’Regan, E. Louise Thomas, Oliver D. Howes

**Affiliations:** 1 MRC London Institute of Medical Sciences and Imperial College London Institute of Clinical Sciences, Hammersmith Campus, Du Cane Road, London, W12 0NN UK; 2 Department of Psychiatry, University of Cambridge, Cambridge, UK; 3 Cambridgeshire and Peterborough NHS Foundation Trust, Cambridge, UK; 4 South London and Maudsley NHS Foundation Trust, London, UK; 5 Imperial College London, London, UK; 6 Institute of Psychiatry, Psychology and Neuroscience, King’s College London, London, UK; 7 Research Centre for Optimal Health, School of Life Sciences, University of Westminster, London, UK

**Keywords:** Schizophrenia, Diagnostic markers, Pathogenesis

## Abstract

Cardiovascular diseases are the leading cause of death in schizophrenia. Patients with schizophrenia show evidence of concentric cardiac remodelling (CCR), defined as an increase in left-ventricular mass over end-diastolic volumes. CCR is a predictor of cardiac disease, but the molecular pathways leading to this in schizophrenia are unknown. We aimed to explore the relevance of *hypertensive* and *non-hypertensive* pathways to CCR and their potential molecular underpinnings in schizophrenia. In this multimodal case–control study, we collected cardiac and whole-body fat magnetic resonance imaging (MRI), clinical measures, and blood levels of several cardiometabolic biomarkers known to potentially cause CCR from individuals with schizophrenia, alongside healthy controls (HCs) matched for age, sex, ethnicity, and body surface area. Of the 50 participants, 34 (68%) were male. Participants with schizophrenia showed increases in cardiac concentricity (*d* = 0.71, 95% CI: 0.12, 1.30; *p* = 0.01), indicative of CCR, but showed no differences in overall content or regional distribution of adipose tissue compared to HCs. Despite the cardiac changes, participants with schizophrenia did not demonstrate activation of the *hypertensive* CCR pathway; however, they showed evidence of adipose dysfunction: adiponectin was reduced (*d* = −0.69, 95% CI: −1.28, −0.10; *p* = 0.02), with evidence of activation of downstream pathways, including hypertriglyceridemia, elevated C-reactive protein, fasting glucose, and alkaline phosphatase. In conclusion, people with schizophrenia showed adipose tissue dysfunction compared to body mass-matched HCs. The presence of non-hypertensive CCR and a dysmetabolic phenotype may contribute to excess cardiovascular risk in schizophrenia. If our results are confirmed, acting on this pathway could reduce cardiovascular risk and resultant life-years lost in people with schizophrenia.

## Introduction

Schizophrenia is a major mental illness with a lifetime prevalence of 1% of the world population, and is amongst the top causes of global disease burden in young adults [1]. Patients with schizophrenia have 2–3 times higher mortality than the general population [2] and up to 15 years shorter life expectancy [3, 4]. Mortality due to natural causes is eight times higher than expected [2], predominantly due to higher prevalence of comorbidities including type 2 diabetes, obesity, and cardiovascular disease (CVD) [5]. CVD is the leading medical cause of death in schizophrenia [6], averaging ~14 life-years lost [7], or 60% of deaths [8].

Previous research has shown evidence for cardiac alterations in people with schizophrenia indicative of concentric cardiac remodelling (CCR) [9], myocardial tissue fibrosis, and/or inflammation [10]. CCR — the increase in the ratio of left ventricular (LV) mass over end-diastolic volume — is one of the strongest predictors of future CVD endpoints such as myocardial infarction, coronary insufficiency, heart failure, and stroke [11], while myocardial fibrosis independently predicts both cardiovascular and all-cause mortality [12]. Therefore, the changes found in chronic schizophrenia might account for part of the additional cardiovascular mortality in schizophrenia.

**Fig. 1:**
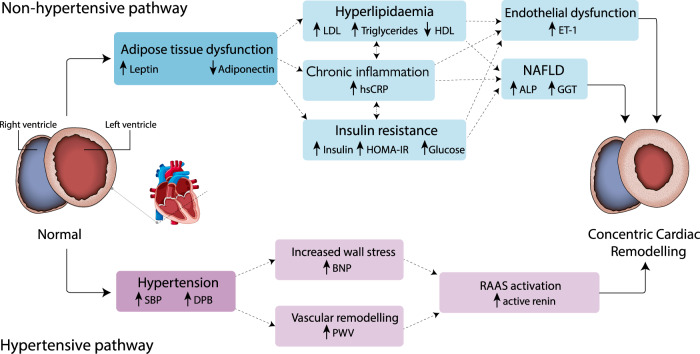
Hypertensive and non-hypertensive molecular pathways to concentric cardiac remodelling. NAFLD non-alcoholic fatty liver disease, ALP alkaline phosphatase, GGT gamma-glutamyltransferase, hsCRP high-sensitivity C-reactive protein, LDL and HDL low- and high-density lipoprotein, HOMA-IR Homeostatic Model Assessment for Insulin Resistance, PWV pulse-wave velocity, BNP brain natriuretic peptide, ET-1 endothelin-1, SBP and DBP systolic and diastolic blood pressure. Graphic element credit: GraphicsRF/Shutterstock.com.

Two main pathological pathways lead to CCR; the most common, the *hypertensive pathway*, is a response to chronic hypertension, both locally on the myocardium causing cardiomyocyte stress, and systemically, as mediated by activation of the renin–angiotensin–aldosterone system, which has potent pro-fibrotic and pro-hypertrophic effects on the myocardium [13] (Fig. 1).

The *non-hypertensive pathway* involves a combination of inflammatory and dysmetabolic changes leading to cardiomyocyte hypertrophy and myo-fibroblast activation [13].

People with chronic schizophrenia have adipose tissue dysfunction, compared to body mass- and fat volume-matched healthy controls (HCs) [14]. Adipose tissue dysfunction is a pro-inflammatory state associated with increased C-reactive protein (CRP) levels [15], insulin resistance [16], and decreased production of adiponectin [16]. Both insulin resistance [14] and low adiponectin [17] have been reported in treated schizophrenia. Reduced adiponectin is associated with a pro-inflammatory state [18, 19] and insulin resistance [19] in the general population. Reduced adiponectin levels are also associated with elevated triglycerides and low-density lipoproteins (LDLs), [20] and with endothelial dysfunction [21].

Figure 1 shows other potential downstream effects of adipose tissue dysfunction, including non-alcoholic fatty liver disease (NAFLD) [22, 23], found to have a higher prevalence in schizophrenia [24].

As inflammation [25], insulin resistance [26], NAFLD [27], hyperlipidaemias [13], and endothelial dysfunction [28] are all independently associated with CCR, adipose tissue dysfunction, with its associated downstream effects, might represent a plausible *non-hypertensive pathway* to CCR in schizophrenia.

The aim of this study was therefore to determine whether there is evidence for the activation of the *hypertensive* or *non-hypertensive* molecular pathways to CCR in schizophrenia.

Given the prior evidence consistent with cardiac inflammation in schizophrenia [10], we hypothesised that there would be lower levels of adiponectin and elevated glucoregulatory and inflammatory markers consistent with activation of the non-hypertensive pathway in patients with schizophrenia.

## Methods

### Participants

Patients with schizophrenia were recruited from South London and the Maudsley NHS Foundation Trust and from Central and North West London NHS Foundation Trust, in the UK. Matched HCs were recruited after matching for age (+/−3 years), ethnicity, sex, and body surface area (BSA +/−0.2, a parameter similar to body mass index (BMI), which is the consensus method do index cardiac parameters [29]; calculated using the Mosteller formula (Height [cm] × Weight [kg]/3600)^½^) through the Hammersmith Hospital Healthy Volunteer Panel, London, UK.

The inclusion criterion for patients was an International Classification of Diseases, Tenth Revision diagnosis of schizophrenia. Exclusion criteria for all participants were: age <18 or >65 years; pregnancy or breastfeeding; a history of cardiometabolic disease, including diabetes, diagnosis of hypertension, dyslipidaemia, ischaemic heart disease, any vascular disorder, other history of congenital/structural cardiac disease; or history of significant or continuing substance abuse and contra-indications to magnetic resonance imaging (MRI). An additional exclusion criterion for HCs was a personal or first-degree family history of schizophrenia or other psychotic disorder.

The patient sample in this study overlaps with our previously published MRI studies, including cardiac [9, 10] and body fat [30] measures by MRI, while HCs show only partial overlap. Further details are in Supplementary Methods.

### Assessment of participants

Physical assessment, study questionnaires, phlebotomy, and MRI were all performed during the same study visit. All patients were assessed at the time of imaging using the Positive and Negative Syndrome Scale for Schizophrenia [31] by a study psychiatrist. Further details are in Supplementary Methods.

### Blood collection and analysis protocol

Blood samples were collected from all participants after at least 6 h of fasting. These were immediately refrigerated, and serum/plasma separation was performed within 30 min. Samples were then frozen at −80 °C for later batch processing. Adipokines (leptin and adiponectin), liver function (alkaline phosphatase (ALP), gamma-glutamyltransferase (GGT), alanine aminotransferase (ALT)), fat metabolism (triglycerides, high-density lipoprotein (HDL) and LDL), inflammation (high-sensitivity CRP (hsCRP)), glucose sensitivity (fasting glucose and insulin), endothelin-1, renin, brain natriuretic peptide, and troponin-I were measured. Processing was performed after maximum 4 years with no intercurrent freeze–thaw cycles. The Homeostatic Model Assessment for Insulin Resistance (HOMA-IR) was calculated as fasting insulin (mIU/l) × fasting glucose (nmol/l)/22.5 [32]. Further details are in Supplementary Methods.

### MRI protocol

MRI was performed at a single site for all subjects, who were recruited between September 2016 and May 2019. Scanning was undertaken on a 3T Siemens Magnetom Prisma (Erlangen, Germany) using a combination of the 18-channel body coil and 12 elements of the 32-channel spine coil. All imaging was acquired with breathing suspended at expiration.

**Fig. 2:**
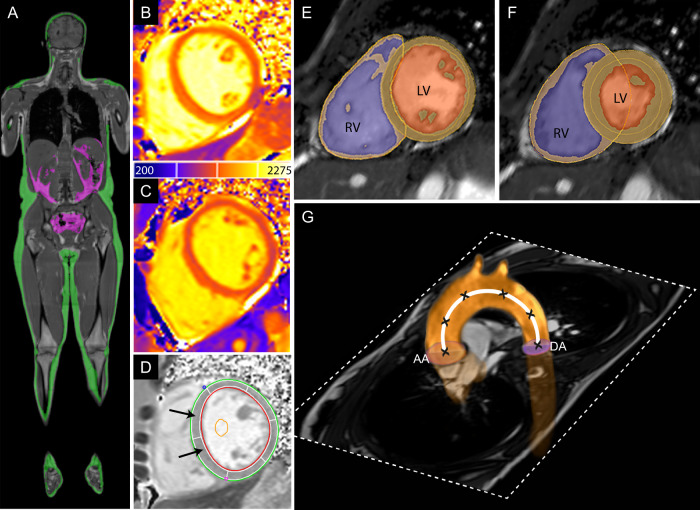
Magnetic resonance images demonstrating the assessment of cardiac function and mass, native T1, pulse-wave velocity, and whole-body fat. **A** Whole body fat. Visceral (green) and subcutaneous (purple) adipose tissue segmentations shown on coronal reformat of whole-body fat image. **B**–**D** Native T1. Representative native T1 map for healthy control (**B**) and a patient with chronic schizophrenia (**C**). Each slice was semi-automatically segmented with endocardial (red) and epicardial (green) borders and divided into six segments (**D**). Native myocardial T1 time was calculated as the mean T1 time the two septal segments (black arrows). **E**, **F** Ventricular function and mass. Endocardial and epicardial contours of the left and right ventricle in diastole (**E**) and systole (**F**). Segmentation shows myocardium (orange), left ventricular (LV) cavity (dark orange), and right ventricular (RV) cavity (purple). **G** Pulse-wave velocity. Transverse cardiac magnetic resonance imaging slice through the aortic arch displaying the magnitude (dotted white slice) with velocity encoded flow through the ascending aorta (AA) and descending aorta (DA). Path length was determined by markers (X) creating a three-dimensional Bezier curve (white line) through the centre of the aorta that intersected the plane at which flow measurements were acquired.

Figure 2 demonstrates the assessment of cardiac function and mass, native T1, pulse-wave velocity (PWV), and whole-body fat through MRI. Further details are in Supplementary Methods, alongside a description of MRI analysis methods.

### Statistical analysis

Data normality was assessed by inspecting distribution plots and tested using Shapiro–Wilk tests. Blood metabolite distributions were skewed to the right and showed a normal distribution after log transformation. Differences among patients and controls were tested using *χ*^2^ tests for categorical variables, Kruskal–Wallis (KW) tests by ranks for non-normally distributed values, and analysis of variance for normally distributed measures. Effect sizes were calculated on normally distributed or transformed data using Cohen’s *d* measure, as implemented in [33].

Logistic regression was used to test for differences in metabolite concentrations after adjusting for smoking, with diagnosis as the outcome and log(metabolite) + smoking status as predictors. To explore any relationship between CCR pathway metabolites and concentricity, linear regression was used to correlate concentricity to the natural logarithm of the metabolite. The overall proportion of variance explained by linear models was calculated using *R*^2^ measures. Further details are in Supplementary Methods.

## Results

The study comprised a total of 50 subjects — 26 patients with treated, chronic schizophrenia and 24 HCs. Two matched HCs were excluded due to poor image quality. Subject characteristics are described in Table 1. At the time of assessment, all patients were treated with antipsychotics (*N* = 23 on clozapine, and *N* = 3 on olanzapine). There were no group differences in age, sex, ethnicity, BSA, physical activity levels, and systolic or diastolic blood pressure (BP). However, patients smoked significantly more than the group controls (Table 1).

**Table 1:** Sample characteristics.

Characteristic	Schizophrenia	Healthy controls	Test statistic
Sample size	26	24	
Male sex, *N* (%)	18 (69%)	16 (67%)	*χ*^2^ = 0.01; df = 1; *p* = 0.99
White ethnicity, *N* (%)	11 (42%)	15 (62%)	*χ*^2^ = 1.3; df = 1; *p* = 0.25
Age, years, mean (SD)	39.9 (10.4)	36.9 (11.4)	*t* = 0.97, df = 47, *p* = 0.34
Number of cigarettes smoked per day, median (min; max; IQR)	5 (0; 35; 18)	0 (0; 10; 0.25)	KW *χ*^2^ = 9.1; df = 1; *p* = 0.003
Smokers, *N* (%)	15 (57.7%)	6 (25%)	*χ*^2^ = 4.2; df = 1; *p* = 0.04
Activity score, median (IQR)	2 (0)	3 (1)	KW *χ*^2^ = 43.8; df = 1; *p* = 0.05
BSA (m^2^), mean (SD)	2.02 (0.30)	1.95 (0.23)	*t* = 0.9, df = 47, *p* = 0.40
Body mass index (BMI) (kg/m^2^), mean (SD)	28.77 (7.19)	25.96 (5.19)	*t* = 1.6, df = 45, *p* = 0.12
Systolic BP (mmHg), mean (SD)	123 (11)	122 (13)	*t* = 0.34, df = 47, *p* = 0.73
Diastolic BP (mmHg), mean (SD)	80 (9)	77 (8)	*t* = 1.1, df = 47, *p* = 0.28
Chlorpromazine equivalent dose (mg/day), median (IQR)	358.98 (221)	N/A	N/A
Duration of treatment (years), median (IQR)	12 (15)	N/A	N/A
Total PANSS score, median (IQR)	55 (29)	N/A	N/A

*df* degrees of freedom, *p* *p* value, *KW χ*^*2*^ Kruskal–Wallis chi squared, *t* *t* test statistic, *SD* standard deviation, *IQR* inter-quartile range, *N/A* not available, *BSA* body surface area (calculated using Mosteller formula), *BP* blood pressure.

### Cardiac and body fat measurements in patients with schizophrenia and matched HCs

Table 2 describes MRI-derived cardiac and fat measures by diagnostic status. Wilk’s test for case–control differences in continuous cardiac measures was significant (Lambda = 0.74, df = 3, *p* = 0.04). The post hoc tests showed that LV concentricity (*d* = 0.71, *p* = 0.01) and native septal T1 (*d* = 0.77, *p* = 0.049) were higher in people with a diagnosis of schizophrenia compared to matched HCs with medium-to-large effect sizes. Mean concentricity in patients was above reference ranges for male adult populations, with 19/26 (73%) of patients and 12/24 (50%) of HCs over the reference range.

**Table 2:** MRI-derived measurements in patients with schizophrenia and matched healthy controls.

	Characteristic	Sample size schizophrenia, HCs	Schizophrenia, mean (SD)	Healthy controls, mean (SD)	Normal range of parameters in males <60 years	One-way MANOVA	Statistical results	Effect size (Cohen’s *d*; 95% CI)
Cardiac measures	Indexed LV mass (g/m^2^)	26, 24	57.17 (19.94)	67.46 (22.37)	57, 91^b^			−0.49; −1.06, 0.09
	Indexed LV EDV (ml/m^2^)	26, 24	64.97 (10.53)	77.67 (12.67)	64, 100^b^			−1.09; −1.70, −0.48
	LV concentricity (g/ml)	26, 24	1.05 (0.22)	0.91 (0.17)	<0.91^b^	Wilk’s Lambda = 0.74, df = 3, *p* = 0.044	*F* (1, 48) = 14.95, *p* = 0.01	0.71; 0.12, 1.30
	Native septal myocardial T1 (ms)	11, 20	1255 (16.2)	1237 (25.1)	N/A		*F* (1, 29) = 4.2, *p* = 0.049	0.77; −0.02, 1.56
	PWV (m/s)	19, 24	5.07 (1.91)	4.22 (1.22)	1.7–8.1^c^		*F* (1, 41) = 3.14, *p* = 0.08	0.54; −0.09, 1.18
Fat measures	Total body fat (l)	20,14	27.90 (15.52)	27.62 (12.62)	N/A	Wilk’s Lambda = 0.98, df = 3, *p* = 0.92	*F* (1, 32) = 0.03, *p* = 0.96	0.02; −0.69, 0.73
	Visceral fat (l)	20,14	3.54 (2.01)	3.74 (1.56)	N/A		*F* (1, 32) = 0.1, *p* = 0.76	−0.11; −0.82, 0.60
	Visceral fat ratio^a^	20,14	0.13 (0.04)	0.14 (0.04)	N/A		*F* (1, 32) = 0.49, *p* = 0.49	−0.24; −0.95, 0.47

Cardiac and fat MRI measurements in patients with chronic schizophrenia and matched healthy controls. Indexed LV mass and EDV were not included in Wilk’s tests or in subsequent testing as their ratio (LV concentricity) is already included but are shown for information.

*LV* left ventricular, *EDV* end-diastolic volume, *PWV* pulse-wave velocity, *df* degrees of freedom, *SD* standard deviation, *CI* confidence interval, *p p* value, *N/A* not available.

^a^Visceral/total body fat.

^b^Based on [53].

^c^Based on [54].

There were no significant differences between groups in PWV (*d* = 0.54, *p* = 0.08), and mean values were within reference ranges for both cases and controls.

Wilk’s test for case–control differences in continuous fat measures was not significant (Lambda = 0.98, df = 3, *p* = 0.92). There was no significant difference in fat volumes (total, visceral and visceral to total ratio) between patients and HCs (Table 2).

### CCR hypertensive pathway in schizophrenia and matched HCs

Wilk’s test for case–control differences in continuous non-hypertensive pathway measures was not significant (Lambda = 0.85, df = 6, *p* = 0.42). There were no differences in either systolic or diastolic BP, PWV (a measure of aortic stiffness, and a proxy for hypertension), active renin levels, N-terminal-prohormone brain natriuretic peptide levels, or troponin I levels (measured to rule out concurrent myocardial damage, e.g. myocarditis) (Supplementary Fig. 1).

### CCR non-hypertensive pathway in schizophrenia and matched HCs

We obtained at least one measure for each step of the non-hypertensive pathway represented in Fig. 1 from our participants. Wilk’s test for case–control differences in continuous non-hypertensive pathway measures was significant (Lambda = 0.35, df = 13, *p* < 0.001).

**Fig. 3:**
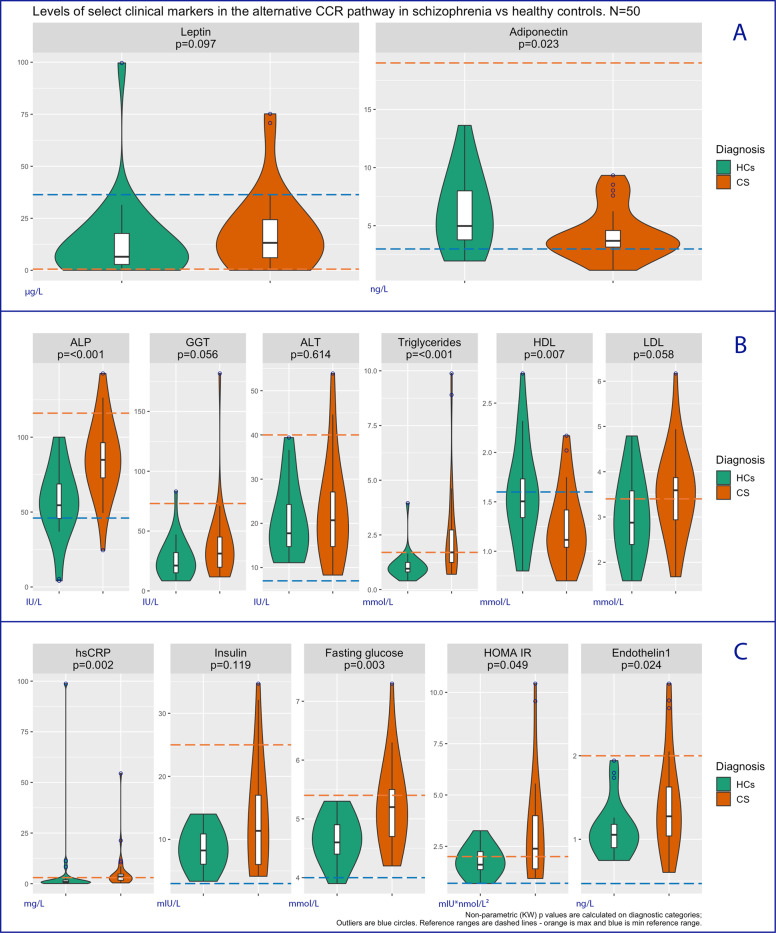
Concentric cardiac remodelling non-hypertensive pathway activity in schizophrenia vs healthy controls. **A** Markers of adipocyte dysfunction. **B** Markers of dyslipidaemia or liver dysfunction (e.g. NAFLD). **C** Markers of inflammation, dysglycaemia, and endothelial dysfunction. *p* Kruskal–Wallis non-parametric test *p* value, HC healthy control, CS chronic schizophrenia, ALP alkaline phosphatase, GGT gamma-glutamyltransferase, ALT alanine aminotransferase, LDL and HDL low- and high-density lipoprotein, hsCRP high-sensitivity C-reactive protein, HOMA-IR Homeostatic Model Assessment for Insulin Resistance.

Figure 3A shows that, among the measures of adipose tissue function, patients had reduced levels of adiponectin (*d* = −0.69, 95% confidence interval (CI): −1.28, −0.10; KW *p* = 0.02) compared to controls; there was no difference in levels of leptin between patients and controls (*d* = 0.25, 95% CI: −0.32, 0.82; KW *p* = 0.10). Patients showed elevated levels of hsCRP (*d* = 0.02, 95% CI: −0.54, 0.59; KW *p* = 0.002; odds ratio for CRP >3 mg/l in patients vs HCs: 5.83, 95% CI: 1.56, 21.87; Fig. 3C) compared to controls. Fasting glucose levels (*d* = 0.96, 95% CI: 0.35, 1.57; KW *p* = 0.003) and the HOMA insulin resistance index (*d* = 0.76, 95% CI: 0.16, 1.36; KW *p* = 0.049) were increased in patients compared to controls (Fig. 3C); however, the difference in fasting insulin levels between the two groups was not statistically significant despite a medium/large effect size (*d* = 0.77, 95% CI: 0.18, 1.36; KW *p* = 0.12). Triglyceride levels were increased in patients compared to controls (*d* = 0.82, 95% CI: 0.23, 1.41; KW *p* < 0.001); HDL-cholesterol levels were reduced in patients (*d* = −0.78, 95% CI: −1.37, −0.19; KW *p* = 0.01). There was no difference in LDL-cholesterol levels between the two groups (*d* = 0.60, 95% CI: 0.02, 1.18; KW *p* = 0.06) (Fig. 3B).

Furthermore, patients showed significant elevations of circulating endothelin-1 levels, a pro-fibrotic, pro-hypertrophic, vasoactive factor released by endothelial cells in response to endothelial stress (*d* = 0.70, 95% CI: 0.11, 1.28; KW *p* = 0.02; Fig. 3C).

Patients showed evidence of elevated ALP (*d* = 1.18, 95% CI: 0.57, 1.80; KW *p* < 0.001), while GGT (*d* = 0.46, 95% CI: −0.12, 1.04; KW *p* = 0.07) and ALT (*d* = 0.22, 95% CI: −0.35, 0.79; KW *p* = 0.61) alterations were not statistically significant (Fig. 3B).

### Sensitivity analysis of significant non-hypertensive pathway metabolites

After adjusting the analysis for smoking, patients still showed reduced levels of log-transformed adiponectin (*z* = −2.1; *p* = 0.03) compared to controls. Further, log-transformed hsCRP (*z* = 2.4, *p* = 0.02), insulin (*z* = 2.2, *p* = 0.03), glucose (*z* = 2.8, *p* < 0.01), and triglyceride (*z* = 2.7, *p* = 0.01) levels were elevated after adjusting for smoking. Finally, log-transformed endothelin-1 levels did not show a significant difference between patients and controls when adjusting for smoking (*z* = 1.4, *p* = 0.15).

### Relationship between CCR and non-hypertensive pathway measures

Simple linear regression was used to test the association between significantly altered non-hypertensive pathway metabolites and participants’ concentricity values.

Table 3 shows that, after adjusting *p* values for multiple testing, only log-transformed adiponectin values showed a significant negative association with cardiac concentricity (*β* = −1.16, *p* = 0.01), explaining 20% of its variance (Supplementary Fig. 2).

**Table 3:** Relationship between cardiac concentricity and non-hypertensive pathway measures in schizophrenia.

	Whole sample					Schizophrenia sample					Supplementary Figs.
	*R*^2^	*F* (48, 1)	*β* for concentricity	*p*	BH *p*	*R*^2^	*F* (24,1)	*β* for concentricity	*p*	BH *p*	
Log(adiponectin)	0.20	11.70	−1.16	0.001	0.01	0.15	4.12	−0.91	0.05	0.378	2
Log(triglycerides)	0.11	6.05	1.07	0.02	0.06	0.07	1.77	0.82	0.20	0.392	3
Log(endothelin-1)	0.10	5.12	0.50	0.03	0.06	0.05	1.38	0.39	0.25	0.392	4
Log(ALP)	0.02	1.22	0.48	0.27	0.38	0.03	0.67	0.26	0.42	0.490	5
Log(HDL)	0.06	3.30	−0.40	0.08	0.13	0.05	1.39	−0.31	0.25	0.392	6
Log(hsCRP)	<0.01	0.08	0.30	0.78	0.78	<0.01	0.06	0.25	0.81	0.810	7
Log(fasting glucose)	0.01	0.60	0.07	0.44	0.51	0.05	1.24	−0.13	0.28	0.392	8

Simple linear regression analysis was used to test the association between significantly altered non-hypertensive pathway measures and participants’ concentricity values.

*ALP* alkaline phosphatase, *hsCRP* high-sensitivity C-reactive protein, *HDL* high-density lipoprotein, *BH* Benjamini and Hochberg.

Log-transformed triglycerides (*β* = 1.07, *p* = 0.02, Supplementary Fig. 3) and endothelin-1 (*β* = 0.50, *p* = 0.03, Supplementary Fig. 4) levels showed a positive association with cardiac concentricity; however, they did not reach the significance threshold after adjusting for multiple testing.

Log-transformed ALP, HDL, hsCRP, and fasting glucose did not show any significant correlation with concentricity measures (Table 3 and Supplementary Figs. 5–8).

Lifetime antipsychotic dose (chlorpromazine equivalents*years) did not show any significant correlation with concentricity measures (*β* < 0.0001, *p* = 0.68) or with log-transformed adiponectin levels (*β* < 0.0001, *p* = 0.81).

## Discussion

In this work, combining cardiac and whole-body fat MRI imaging measures with blood assessment of various metabolic and inflammatory markers, we investigate several molecular pathways potentially responsible for CCR in chronic schizophrenia. We show evidence of adipose tissue dysfunction in schizophrenia, as well as downstream effects that might explain up to 20% of the CCR phenotype, in the absence of case–control differences in age, sex, ethnicity, BSA, body fat total content and distribution, and any medical comorbidity.

We have previously shown that patients with chronic schizophrenia show evidence of CCR [9] and increased myocardial T1 relaxation time, which is evidence of inflammation/fibrosis [10]. We have also shown that patients with chronic schizophrenia do not demonstrate differences in body fat content or distribution as compared to matched HCs [30].

We confirm these findings in the present, partially overlapping sample, with participants additionally tested for a number of blood metabolites from the hypertensive and non-hypertensive CCR pathways. Here we also show that CCR in people with schizophrenia might have a different aetiology from that of the general population, i.e. it does not appear to be associated with the most common pathological stimuli, such as pressure overload (no difference in systolic or diastolic BP in schizophrenia vs HC), which usually leads to stiffening of the aorta (PWV not significantly elevated), and activation of the renin–angiotensin axis (no active renin elevation).

Despite no differences in body fat content or distribution, we show that patients with schizophrenia do manifest adipose tissue dysfunction, as evidenced by low adiponectin levels. Low levels of adiponectin are associated with a more atherogenic lipid profile, including increases in triglycerides and LDL, and reductions in HDL [20]; a similar pattern of association suggestive of adiponectin effects was present in this study but will require confirmation in larger, better powered samples. Previous evidence supports our findings by suggesting that adiponectin, leptin, and their ratio might be useful prognostic markers of metabolic syndrome in schizophrenia [34].

Further, a dyslipidaemic phenotype has been associated with chronic inflammatory states [18, 19], and indeed we show significant elevations in CRP in our sample, which has been associated with NAFLD [22]. Although we did not measure liver fat content directly in this study, we found significantly elevated levels of ALP, a liver function marker, and triglycerides, suggestive of NAFLD. These findings agree with reports in the literature that the prevalence of NAFLD is higher in patients with schizophrenia than in the wider population [35]. As adiponectin has been shown to decrease in response to smoking in the general population [36], we were also able to show that low adiponectin levels in schizophrenia appear independent of the increased rate of smoking in this patient population.

Finally, there are known causal associations between low adiponectin and insulin resistance [19], which we show evidence of in schizophrenia, as well as between low adiponectin and endothelial dysfunction [21], and indeed we find that endothelin-1 is significantly elevated in schizophrenia.

We also demonstrate a negative correlation between adiponectin levels (the first step in the pathway) and concentricity values (the end result), which supports a potentially causal role. Further, our analysis shows that one-fifth (20%) of the variance of concentricity is explained by adiponectin levels in our sample.

### Implications of our findings

We have recently shown that schizophrenia is characterised by CCR [9], which could explain part of the additional cardiovascular mortality in schizophrenia, as CCR has been shown to be associated with cardiovascular events in healthy adults [11].

By combining blood and imaging measures, in this study we take a step forward, by showing that adipose tissue dysfunction, and particularly reductions in adiponectin, may play a role in orchestrating multiple dysmetabolic changes in schizophrenia, independently of changes in BP. We did not find case–control differences in leptin; however, as leptin is usually a reflection of subcutaneous fat content, this lack of a significant difference is in agreement with our findings of no differences in body fat content or distribution in patients with schizophrenia.

Adiponectin is secreted by adipocytes, decreasing when fat mass increases, such as in obesity or metabolic syndrome, in a negative feedback loop [37]. Dysfunctional adipocytes produce lower levels of adiponectin but higher levels of pro-inflammatory cytokines, which further inhibit the production of adiponectin in adipocytes. Adiponectin expression by adipocytes is also inhibited by oxidant stress [38]. Adiponectin levels are strongly negatively correlated with CVD: adiponectin concentrations in the plasma are reduced in coronary artery disease (CAD) patients, compared to age- and BMI-adjusted control subjects [39], and high adiponectin levels are associated with lower risk of CAD in healthy men [40]. Finally, adiponectin reductions are associated with increased CRP and other pro-inflammatory cytokines [18, 19], insulin resistance [19], elevated plasma triglycerides and lipoproteins [20], and endothelial dysfunction [21] in the general population. As inflammation [25], insulin resistance [26], NAFLD [27], hyperlipidaemias [13], and endothelial dysfunction [28] can all independently trigger CCR, adipose tissue dysfunction, with its associated downstream effects, might represent a plausible non-hypertensive pathway to CCR in schizophrenia.

We therefore postulate that schizophrenia may be characterised by adipose tissue dysfunction, and this may contribute to CCR and increases in the risk of CVD, and ultimately in additional mortality. If confirmed in larger prospective studies, these findings would suggest that reparative and preventative strategies could be aimed at restoring balance to the adipose tissue system, even in the absence of macroscopic changes to the body’s fat mass.

### Strengths and limitations

The main strength of this study is the integration of multiple methods and investigation techniques, including clinical phenotypes, cardiac and fat MRI, and blood markers, all in the same sample. The integration of imaging and biochemical sampling in a select population with severe mental illness did constrain the number of participants that we could enrol and consequently sample size. It is therefore possible that some of the negative findings in this work might be the result of a type II statistical error.

A further strength is represented by the use of gold standard techniques for measuring cardiac, body fat, and blood phenotypes, allowing all measurements to be analysed by operators blind to diagnosis. For cardiac phenotypes, we utilised cardiovascular magnetic resonance (CMR), which is the gold standard for LV function and mass quantification [29]. A key advantage of CMR over echocardiography is that the ventricles can be imaged in their entirety, and no geometrical assumptions need to be made in order to derive global whole-organ data [41]. Body fat contents and distribution was measured using gold standard whole-body MRI, which has been shown to outperform other techniques such as body impedance by not providing underestimates of body mass overall [42], and more accurate quantification of the visceral compartment [43].

A third major strength of this study is that we excluded any participants with a pre-existing medical condition, including heart disease, hypertension, diabetes, or dyslipidaemia, and we matched patients and controls for BSA, age, sex and ethnicity, all potential confounders in analyses of cardiometabolic function [29, 44–46]. However, while we excluded diagnosed cardiometabolic disorders, we cannot exclude the possibility of subclinical changes, although we found no significant difference between cases and controls in systolic or diastolic BP or PWV, suggesting subclinical differences are unlikely to be large.

The main limitation of this study is that it involved chronic patients (median duration of treatment of 12 years), who were all taking metabolically active antipsychotic medications, mainly clozapine. Olanzapine and clozapine in particular are known to have dysmetabolic effects [47]. It is therefore possible that adipose tissue dysfunction in schizophrenia is mediated at least in part by their use. Bartoli et al. found that adiponectin may be significantly reduced in chronic patients taking second-generation antipsychotics (SGAs), with non-significant findings in a smaller sample of antipsychotic-free patients [17]. Multiple reports associate treatment with olanzapine or clozapine (not risperidone) with adiponectin reductions [17, 48], suggesting that the effect may be medication specific. However, in the current study, where all participants were chronic patients taking SGA, mostly clozapine, we did not find a linear relationship between total lifetime chlorpromazine equivalents and either concentricity or adiponectin levels. In any respect, a future study in antipsychotic-naive patients, who already show pro-inflammatory [49] and dysmetabolic changes [50], would be useful to determine whether the cardiac and adipokine changes we have detected are already present in psychosis before treatments are started.

A final limitation is the absence of liver imaging or spectroscopy to directly assess liver fat content; however, we relied on a panel of blood tests to indirectly assess the risk of NAFLD.

## Conclusions and future directions

In participants with chronic schizophrenia with evidence of CCR, we found no differences in adipose tissue content or regional distribution or hypertension compared to well-matched HCs, despite reductions in adiponectin. We hypothesise that cardiac changes may originate from adipose tissue dysfunction and its downstream effectors, potentially mediated by significant reductions in adiponectin.

The role of adipose tissue dysfunction in schizophrenia, and in particular the role of circulating adiponectin levels, could provide a useful biomarker to classify cardiovascular risk in patients with schizophrenia, as it has already shown to have potential as a predictor of developing metabolic syndrome in this same cohort [51, 52]. Additionally, it raises the potential for interesting therapeutic avenues to reduce cardiovascular risk in certain patients.

Adiponectin levels in schizophrenia need further investigation, and the preliminary finding that they are reduced in chronic patients on SGAs [17] should encourage further research on the effects of specific antipsychotics on adipose tissue function.

## Supplementary information

Supplementary information
