# Supplementary material for: Adipose tissue dysfunction, inflammation, and insulin resistance: alternative pathways to cardiac remodelling in schizophrenia. A multimodal, case–control study
Source: Transl Psychiatry. 2021 Dec 6;11:614. doi: 10.1038/s41398-021-01741-9 (PMC8648771; doi:10.1038/s41398-021-01741-9)
Supplement: Supplementary file 1 — Supplementary information [file 41398_2021_1741_MOESM1_ESM.pdf]

Osimo et al, 2021
Adipose tissue dysfunction, inflammation, and insulin resistance: alternative pathways to cardiac remodelling in schizophrenia.
A multimodal, cross-sectional study

Supplementary materials

Table of contents

Supplementary methods **2**
- Participants 2
- Assessment of participants 2
- Blood assays 2
- Magnetic Resonance Imaging 3
- MR Image Analysis 4
- Statistical analysis 5

Supplementary figures **6**

Additional references **10**

Supplementary methods

Participants

All participants underwent cardiac, whole body magnetic resonance imaging (MRI) and venepuncture. However, native T1 time was only acquired for a subset of later participants due to an intercurrent protocol change.
Written informed consent was obtained from all volunteers. All procedures contributing to this work comply with the ethical standards of the relevant national and institutional committees on human experimentation and with the Helsinki Declaration of 1975, as revised in 2008. All procedures involving human subjects were approved by the London - Camberwell St Giles Research Ethics Committee.

Assessment of participants

Participants were seated in a quiet, temperature-controlled waiting room and given time to relax. A doctor then performed a screening interview, clinical examination, and supervised the MRI. The presence of medical co-morbidities was assessed directly by interviewing participants, by examining the list of active prescriptions, and where in doubt via examining general practice records. Brachial blood pressure (BP) measurement was performed following 5 min rest using a validated oscillometric device (Omron M7, Omron Corporation, Kyoto, Japan). The first of 3 measures were discarded, and the second 2 values were averaged to provide the final reading. Physical activity grading was based on the Copenhagen City Heart Study Leisure Time Physical Activity Questionnaire, performed on the same day as the cardiac magnetic resonance imaging. Categories of activity were based on participants' level of activity over the preceding 12 months, ranging from level 1: almost entirely sedentary, to level 4: >5 hours of exercise per week (Dawes, Corden et al. 2016). Antipsychotic doses were converted into chlorpromazine equivalents as described by Andreasen and colleagues (Andreasen, Pressler et al. 2010).

Blood assays

All blood analyses were performed using pseudonymised participant codes, which preserved the operator's blinding to diagnosis.

Adiponectin was measured by Quantikine Adiponectin ELISA kit, catalogue number DRP300, distributed by Biotechne, R & D Systems Europe, 19 Barton Lane, Abingdon Science Park, Abingdon, Oxon, OX14 3NB. Sensitivity >0.246 μg/L. Linearity up to 250 μg/L.
Leptin was measured by Quantikine Leptin ELISA kit, catalogue number DLP00, distributed by Biotechne (R & D Systems Europe), 19 Barton Lane, Abingdon Science Park, Abingdon, Oxon, OX14 3NB. Sensitivity >7.8 ng/L. Linearity up to 1000 ng/L.
Alkaline phosphatase was measured by the ADVIA Chemistry alkaline phosphatase (ALP-2) assay, catalogue number 10916067, distributed by Siemens Healthcare Diagnostics Ltd, Newton House, Sir William Siemens Square, Frimley, Camberley, Surrey GU16 8QD. Sensitivity >10 IU/L. Linearity up to 1000 IU/L.
Gamma glutamyl transferase (GGT) was measured by the SIEMENS ADVIA 1800 GAMMA GLUTAMYL TRANSFERASE assay, reagent catalogue number 07498649 is distributed by Siemens Healthineers Ltd, Newton House, Sir William Siemens Square, Frimley, Camberley, Surrey GU16 8QD. Sensitivity down to 0 IU/L. Linearity up to 1200 IU/L.
Alanine aminotransferase was measured by The ADVIA Chemistry Alanine Aminotransferase (ALTP5P) method, reagent catalogue number 07501976 is distributed by Siemens Healthineers Ltd,

Newton House, Sir William Siemens Square, Frimley, Camberley, Surrey GU16 8QD. Sensitivity >5.0 IU/L. Linearity up to 1000 IU/L.
Triglycerides were measured by the ADVIA Chemistry Triglyceride assay, catalogue number 10335892 is distributed by Siemens Healthineers Ltd, Newton House, Sir William Siemens Square, Frimley, Camberley, Surrey GU16 8QD. Sensitivity >0.11 mmol/L. Linearity up to 6.22 mmol/L.
HDL cholesterol was measured by the Siemens Advia 1800 HDL assay, catalogue number 07511947 is distributed by Siemens Healthineers Ltd, Newton House, Sir William Siemens Square, Frimley, Camberley, Surrey GU16 8QD. Sensitivity >0.1 mmol/L. Linearity up to 3.0 mmol/L.
LDL cholesterol was measured by the Siemens Advia 1800 LDL assay, catalogue number 09796248 is distributed by Siemens Healthineers Ltd, Newton House, Sir William Siemens Square, Frimley, Camberley, Surrey GU16 8QD. Sensitivity >0.09 mmol/L. Linearity up to 25.9 mmol/L.
High sensitivity C reactive protein (CRP) was measured by the ADVIA Chemistry Cardiophase high sensitivity C reactive protein assay, reagent catalogue number 06837459 is distributed by Siemens Healthineers Ltd, Newton House, Sir William Siemens Square, Frimley, Camberley, Surrey GU16 8QD. Sensitivity >0.16 mg/L. Linearity up to 10.0 mg/L; Siemens have validated an automatic rerun condition for this method that extends the reportable range up to 200.0 mg/L.
Insulin was measured by the SIEMENS CENTAUR XP INSULIN assay, catalogue number 2230141 supplied by Siemens Healthcare Diagnostics Ltd, Newton House, Sir William Siemens Square, Frimley, Camberley, Surrey GU16 8QD. Sensitivity >0.5 mIU/L. Linearity up to 300 mIU/L.
Plasma glucose was measured by the hexokinase / G-6-PDH Abbott assay. Linearity range 0.28 – 44.4 mmol/L.
Endothelin-1 was measured by the Quantikine Endothelin-1 ELISA kit, catalogue number DET100, distributed by Biotechne, R & D Systems Europe, 19 Barton Lane, Abingdon Science Park, Abingdon, Oxon, OX14 3NB. Sensitivity >0.087 ng/L. Linearity up to 25 ng/L.

Magnetic Resonance Imaging

Cardiac MRI Acquisition

Ventricular function was assessed in the conventional manner using balanced steady-state free precession (bSSFP) cine images acquired in cardiac short-axis and long-axis planes (Kramer, Barkhausen et al. 2020) using a repetition time (TR), 3.3ms; echo time (TE), 1.43ms, flip angle, 40°; voxel size 1.5x1.5x8mm; bandwidth 962Hz/pixel; parallel imaging factor, 3; and temporal resolution, 45ms.
Pulse wave velocity was assessed using a phase contrast (PC) sequence positioned perpendicular to both the ascending and descending aorta at the level of the bifurcation of the pulmonary trunk. The PC data were acquired with a retrospectively ECG-gated gradient echo sequence using a velocity encoding gradient of 180cm/s in the through-plane direction and a TR of 4.6ms; TE, 2.47ms, flip angle, 20°; voxel size 1.8x1.8x6mm; bandwidth 949Hz/pixel; parallel imaging factor, 2; and temporal resolution, 37ms. To calculate path length, a bSSFP single-shot sequence was acquired in a sagittal plane through the aortic arch using a TR of 3ms, TE, 1.23ms, flip angle, 60°; voxel size 1.3x1.3x8mm; bandwidth 849Hz/pixel; parallel imaging factor, 2.
Myocardial tissue changes were assessed according to consensus guidelines (Messroghli, Moon et al. 2017) by measuring the longitudinal relaxation time constant (native myocardial T1 time). A Modified Look-Locker Inversion recovery (MOLLI) sequence, 5s (3s) 3s version, was acquired in a mid-ventricular short axis slice using a prospectively ECG-gated bSSFP single-shot sequence and a TR of 2.7ms; TE, 1.12ms, flip angle, 35°; voxel size 1.4x1.4x8mm; bandwidth 1085Hz/pixel; parallel imaging factor, 2.

Whole Body MR Acquisition

To assess whole-body fat, participants were positioned supine in the scanner and were scanned from the top of their head to their toes. Axial images were acquired using the integrated body coil in 10 contiguous sections of sixty slices with a Dixon volumetric interpolated breath-hold examination (VIBE) sequence and a TR of 6.53ms, TEs, 1.34ms and 2.57ms, flip angle, 10°; voxel size 2.5x2.5x3mm; bandwidth 620Hz/pixel. Sections through the neck, chest and abdomen were acquired at suspended expiration.

MR Image Analysis

All MR image analysis was performed using pseudonymised participant codes, which preserved the operator's blinding to diagnosis.

Cardiac Mass and Volume

Volumetric analysis of the cine images was performed using CMRtools (Cardiovascular Imaging Solutions, London, UK) by an experienced user (10 years of CMR experience), blind to diagnosis. Subjects whose images were degraded by respiration or ECG synchronisation artefacts to the extent where cardiac contours could not be clearly identified were excluded from the analysis. After manual segmentation of epi- and endocardial borders at end-diastole and end-systole, semi-automated thresholding was used to identify the papillary muscles; these were included in the left ventricular mass and excluded from volumetric measurements. To increase the accuracy of measurements, the valve positions were identified on the long-axis images, allowing the valve planes to be tracked through the cardiac cycle.
Volumes and mass were indexed to body surface area to give the indexed volumetric data: left ventricular mass (LVMi) and left ventricular end-diastolic volumes (LVEDVi). The end-diastolic volume was calculated using the Simpson's method of discs(Schiller, Shah et al. 1989).

Whole body fat MRI

Fat content and distribution were determined as previously described(O'donovan, Thomas et al. 2009). Images were analysed using SliceOmatic (Tomovision, Montreal, Quebec, Canada), and regional volumes were recorded in litres (L), including total adipose tissue and visceral fat(Thomas, Parkinson et al. 2012).

Pulse Wave Velocity

PWV analysis was performed using the ARTFUN software (ART-FUN; Inserm, Paris, France) (Dogui, Redheuil et al. 2011) by an experienced CMR user (6 years CMR experience). Using a magnitude PC image, the contours of the ascending and proximal descending aortic were semi-automatically delineated and then propagated throughout the cardiac cycle. To define the path length, six to eight markers were defined across the aortic arch to create a three-dimensional Bezier curve through the centre of the aorta that intersected the plane where the flow measurements were acquired. The arch-PWV was calculated as the ratio between this 3D length of the aortic arch, and the transit time (Dt) between the velocity waveforms in the ascending and descending aorta.

Native T1

The MOLLI sequence was analysed using Circle Cardiovascular Imaging (CVI), Calgary, Canada, version 5.12.1 T1 mapping software. A Siemens recommended correction factor of 1.035 was applied. Each slice was divided into 6 segments, as per the American Heart Association model, with the 2 septal segments defined by the anterior and inferior right ventricular insertion points. An epicardial and endocardial erosion offset of 10% was applied to the contours to ensure only

myocardium was included. Septal T1 was derived by taking a mean of the two septal segments; septal T1 was used in place of the global T1 of the mid-ventricular short axis slice, to reduce partial volume artefact.

Statistical analysis

For measures such as CRP, which have an established risk cutoff, odds ratios (ORs) and 95% confidence intervals (CIs) for "high" vs "low" values in schizophrenia vs controls were calculated and reported separately. All multivariable analyses use continuous values.
All analyses were performed in R (R Core Team 2021).
Significance was taken as $p < 0.05$ (two-tailed). Difference in pathways between patients and HCs, were tested using a one-way multivariate analysis of variance (one-way MANOVA or Wilk's test(Todorov and Filzmoser 2009)) for each group of continuous dependent variables, with diagnosis as the independent variable, to determine for group differences. The cardiac measures pathway included LV concentricity, native septal T1, PWV. LVMi and LVEDVi are reported in Table 2 but not included in group testing as already included as their ratio, LV concentricity. The fat measures pathway included total, visceral body fat and their ratio. The hypertensive pathway included systolic and diastolic blood pressure, PWV, active renin, NT-proBNP and Troponin I. The non-hypertensive pathway measures included leptin and adiponectin, ALP, GGT, ALT, triglycerides, HDL and LDL, hsCRP, insulin, glucose, HOMA and endothelin-1. If the Wilk's test was significant, post-hoc tests for each variable were conducted. P values outside of pathways, i.e., linear regression results, were adjusted using the Benjamini & Hochberg method.

Supplementary figures

Supplementary Figure 1: CCR hypertensive pathway activity in schizophrenia vs healthy controls.

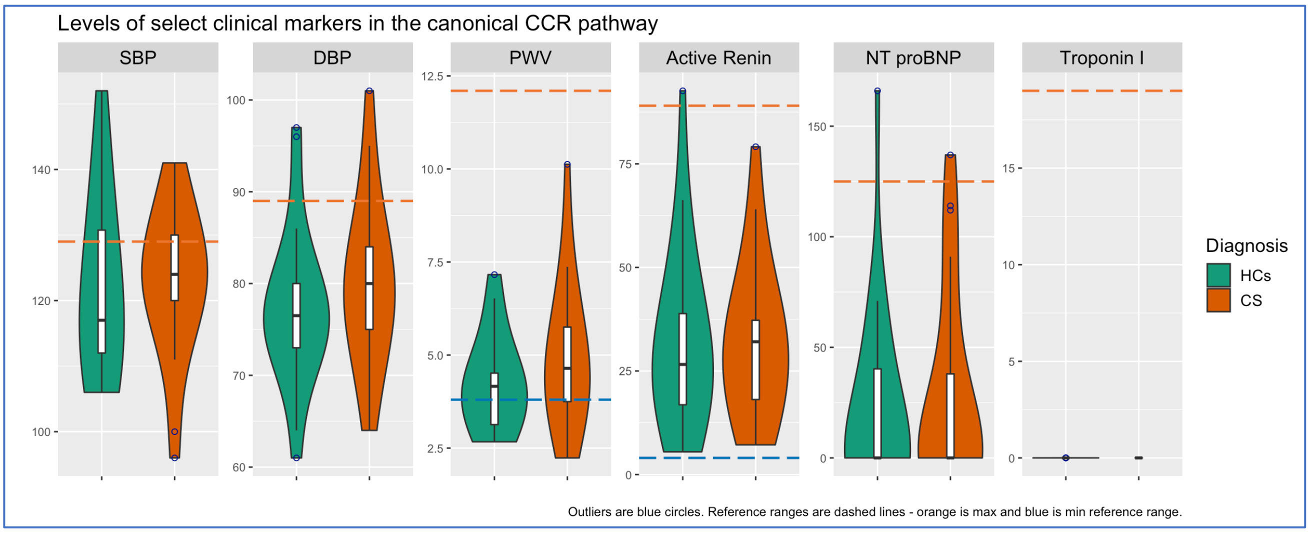

HCs: healthy controls; CS: schizophrenia; SBP and DBP: systolic/diastolic blood pressure; PWV: pulse-wave velocity; NT-pro BNP: n-terminal pro-brain natriuretic peptide

Supplementary Figure 2: regression of log-transformed adiponectin over concentricity in schizophrenia vs healthy controls.

Left panel: whole sample. Right panel: schizophrenia (SCZ) in orange, healthy controls (HC) in green.

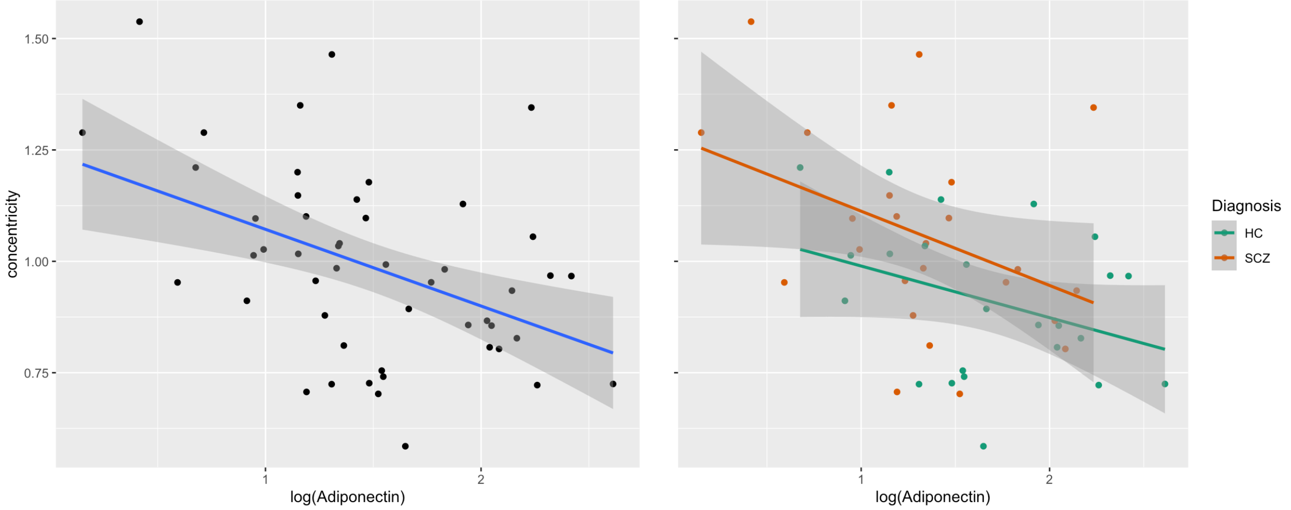

Supplementary Figure 3: regression of log-transformed triglycerides over concentricity in schizophrenia vs healthy controls.

Left panel: whole sample. Right panel: schizophrenia (SCZ) in orange, healthy controls (HC) in green.

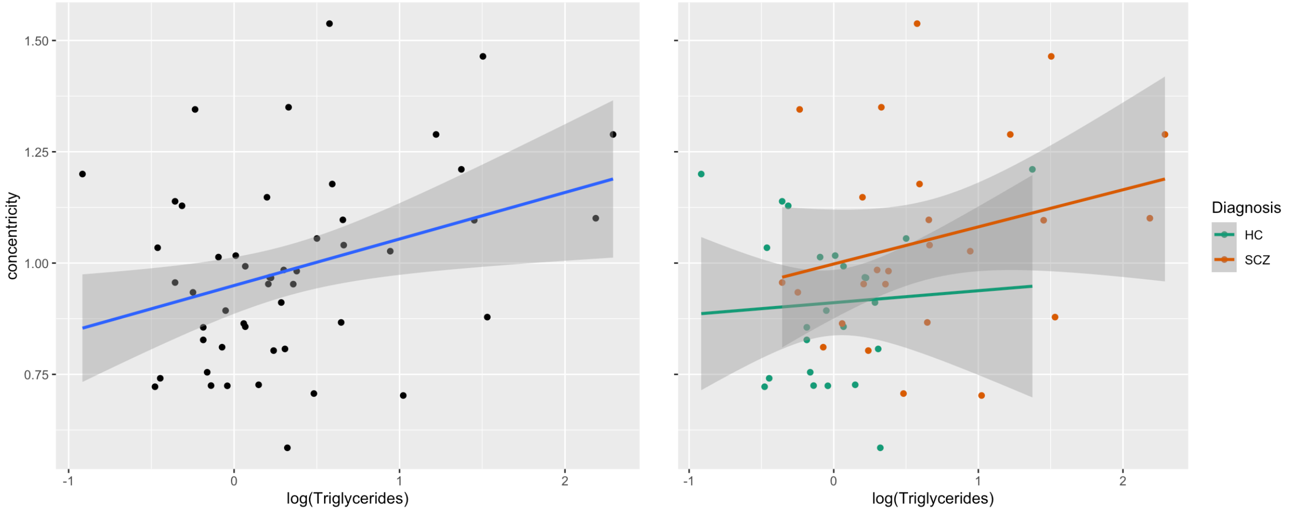

Supplementary Figure 4: regression of log-transformed endothelin-1 over concentricity in schizophrenia vs healthy controls.

Left panel: whole sample. Right panel: schizophrenia (SCZ) in orange, healthy controls (HC) in green.

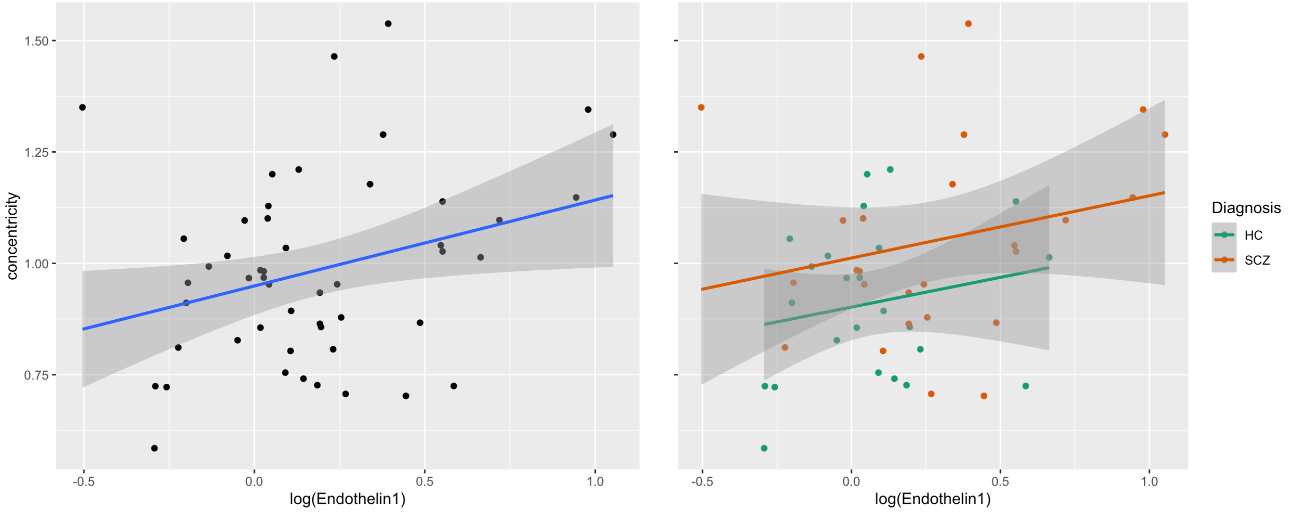

Supplementary Figure 5: regression of log-transformed alkaline phosphatase over concentricity in schizophrenia vs healthy controls.

Left panel: whole sample. Right panel: schizophrenia (SCZ) in orange, healthy controls (HC) in green.
ALP: alkaline phosphatase

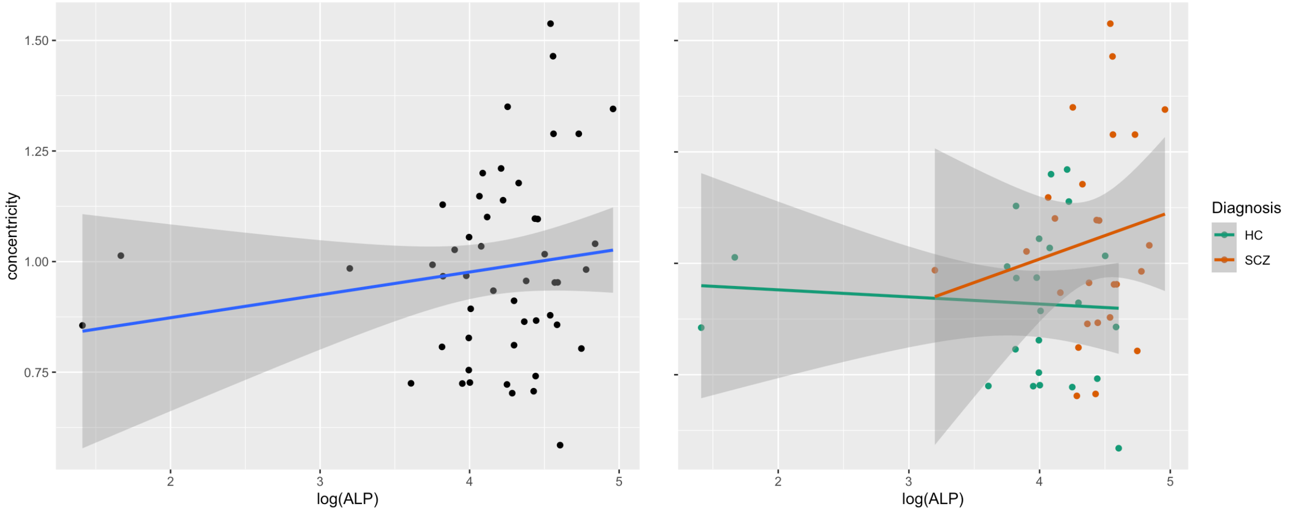

Supplementary Figure 6: regression of log-transformed HDL over concentricity in schizophrenia vs healthy controls.

Left panel: whole sample. Right panel: schizophrenia (SCZ) in orange, healthy controls (HC) in green.
HDL: high-density lipoprotein

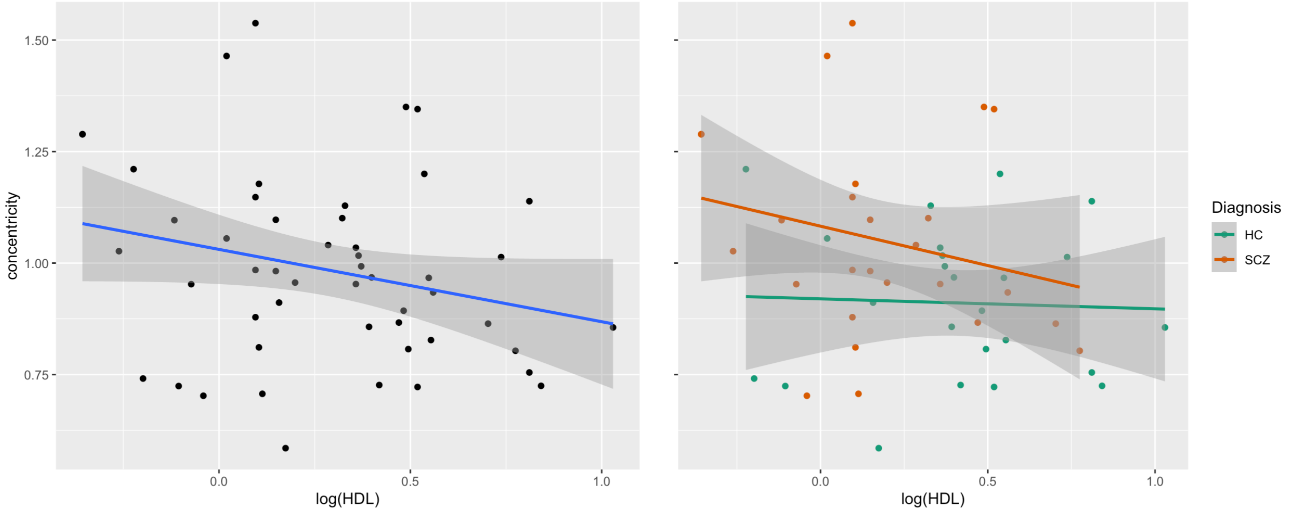

Supplementary Figure 7: regression of log-transformed high-sensitivity CRP over concentricity in schizophrenia vs healthy controls.

Left panel: whole sample. Right panel: schizophrenia (SCZ) in orange, healthy controls (HC) in green.
hsCRP: high-sensitivity C-reactive protein

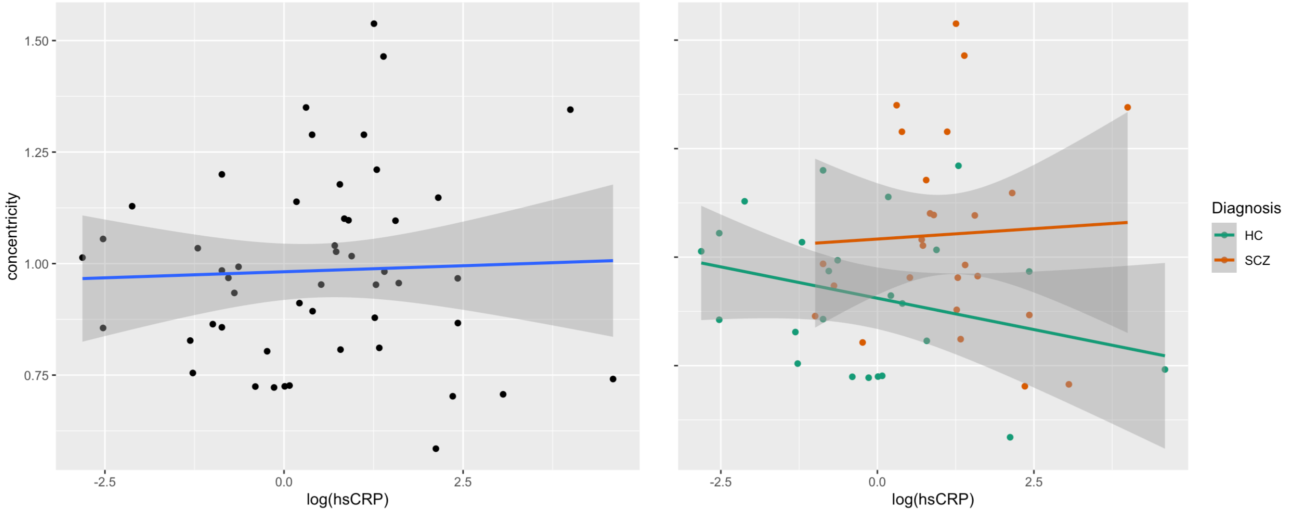

Supplementary Figure 8: regression of log-transformed fasting glucose over concentricity in schizophrenia vs healthy controls.

Left panel: whole sample. Right panel: schizophrenia (SCZ) in orange, healthy controls (HC) in green.

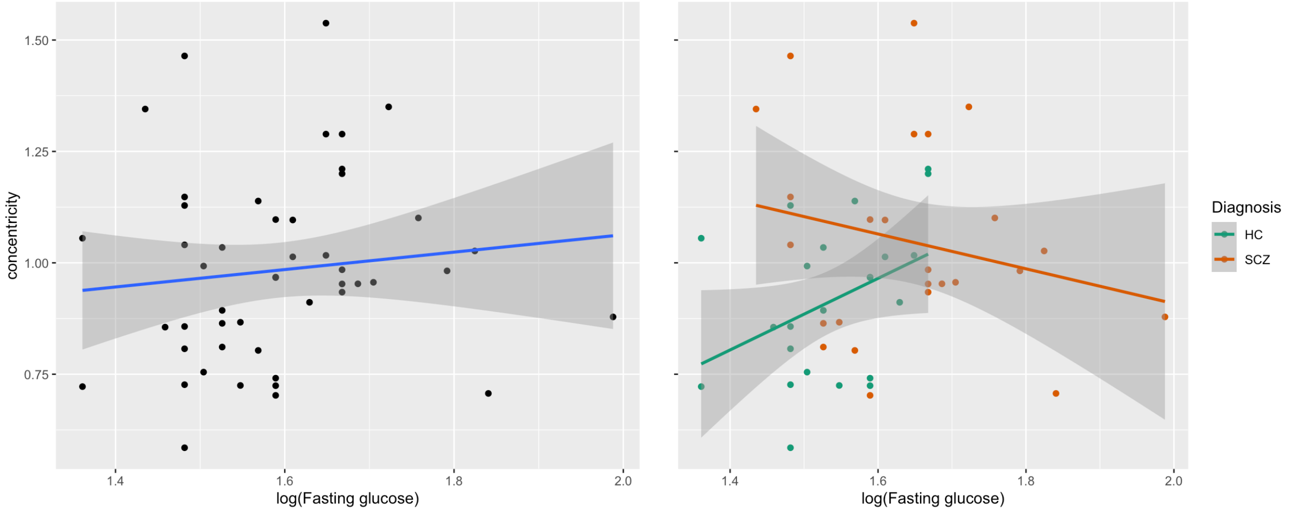

Additional references

Andreasen, N. C., M. Pressler, P. Nopoulos, D. Miller and B. C. Ho (2010). "Antipsychotic Dose Equivalents and Dose-Years: A Standardized Method for Comparing Exposure to Different Drugs." Biological Psychiatry **67**(3): 255-262.

Dawes, T. J., B. Corden, S. Cotter, A. de Marvao, R. Walsh, J. S. Ware, S. A. Cook and D. P. O'regan (2016). "Moderate physical activity in healthy adults is associated with cardiac remodeling." Circulation: Cardiovascular Imaging **9**(8): e004712.

Dogui, A., A. Redheuil, M. Lefort, A. DeCesare, N. Kachenoura, A. Herment and E. Mousseaux (2011). "Measurement of aortic arch pulse wave velocity in cardiovascular MR: comparison of transit time estimators and description of a new approach." Journal of Magnetic Resonance Imaging **33**(6): 1321-1329.

Kramer, C. M., J. Barkhausen, C. Bucciarelli-Ducci, S. D. Flamm, R. J. Kim and E. Nagel (2020). "Standardized cardiovascular magnetic resonance imaging (CMR) protocols: 2020 update." Journal of Cardiovascular Magnetic Resonance **22**(1): 1-18.

Messroghli, D. R., J. C. Moon, V. M. Ferreira, L. Grosse-Wortmann, T. He, P. Kellman, J. Mascherbauer, R. Nezafat, M. Salerno and E. B. Schelbert (2017). "Clinical recommendations for cardiovascular magnetic resonance mapping of T1, T2, T2* and extracellular volume: a consensus statement by the Society for Cardiovascular Magnetic Resonance (SCMR) endorsed by the European Association for Cardiovascular Imaging (EACVI)." Journal of Cardiovascular Magnetic Resonance **19**(1): 1-24.

O'donovan, G., E. L. Thomas, J. McCarthy, J. Fitzpatrick, G. Durighel, S. Mehta, S. Morin, A. P. Goldstone and J. D. Bell (2009). "Fat distribution in men of different waist girth, fitness level and exercise habit." International journal of obesity **33**(12): 1356-1362.

R Core Team (2021). R: A language and environment for statistical computing [Software]. Vienna, Austria, R Foundation for Statistical Computing.

Schiller, N. B., P. M. Shah, M. Crawford, A. DeMaria, R. Devereux, H. Feigenbaum, H. Gutgesell, N. Reichek, D. Sahn and I. Schnittger (1989). "Recommendations for quantitation of the left ventricle by two-dimensional echocardiography." Journal of the American Society of Echocardiography **2**(5): 358-367.

Thomas, E. L., J. R. Parkinson, G. S. Frost, A. P. Goldstone, C. J. Doré, J. P. McCarthy, A. L. Collins, J. A. Fitzpatrick, G. Durighel and S. D. Taylor-Robinson (2012). "The missing risk: MRI and MRS phenotyping of abdominal adiposity and ectopic fat." Obesity **20**(1): 76-87.

Todorov, V. and P. Filzmoser (2009). "An object-oriented framework for robust multivariate analysis." Journal of Statistical Software **32**(1): 1-47.
